# Response to ‘International trends in male youth suicide and suicidal behaviour’

**DOI:** 10.1017/neu.2025.10048

**Published:** 2025-12-17

**Authors:** Chi Chen, Chun-I Liu

**Affiliations:** 1 Department of Addiction Sciences, Taipei City Psychiatric Center, Taipei City Hospital, Taipei, Taiwan; 2 Department of Psychiatry, National Taiwan University Hospital, Taipei, Taiwan

**Keywords:** Suicide, Youth mental health, Substance abuse, Epidemiology, Psychiatry emergency

To the Editor,

We read with great interest the recent article by Rice et al., titled ‘International trends in male youth suicide and suicidal behaviour’. The study provides a valuable summary of suicide trends across different countries and highlights the growing crisis of substance abuse among male youth. However, the article did not include data from Taiwan. In this response, we present suicide trends from Taiwan and extend the discussion by providing quantitative analyses of diagnostic patterns and sex-specific differences in psychiatric emergency visits, which maybe correlated with the higher suicide rates observed among male youth.

Our research demonstrates that the global increase in suicide rates and the prevalence of psychiatric disorders is mirrored in Taiwan’s national data. Suicide rates have risen significantly since 2015 (from 15.1 per 100 000 in 2014 to 16.7 in 2023) (Chang et al., 2023). According to national suicide surveillance data from 2015 to 2023, suicide mortality increased by 400% among individuals aged 0–14 years and by 142% among those aged 15–24 years. Reported suicide risk cases rose even more dramatically – by 888% in the 0–14 age group and 302% in the 15–24 age group – while increases were less pronounced in older populations (116% in the 25–44 group, 118% in the 45–64 group, and 174% in those >65 years) (Supplementary Figure 1).

**Figure 1.**
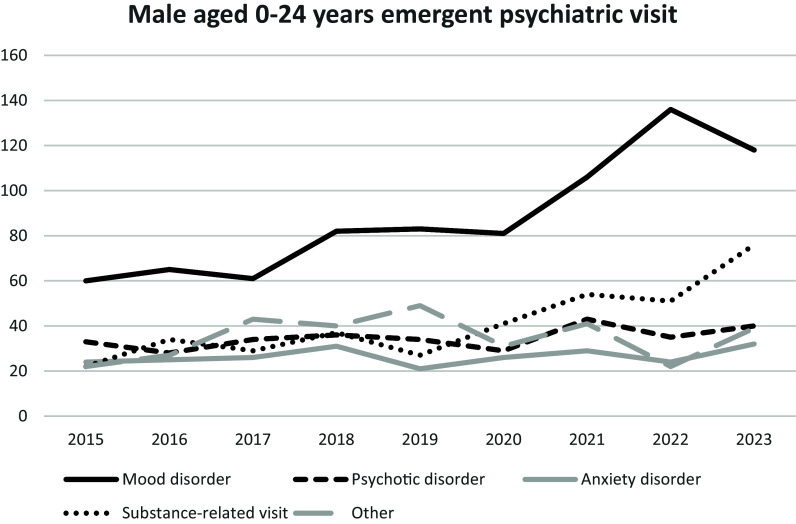
Single-centre psychiatric emergency visits, grouped by tentative diagnosis. Diagnoses were established through a 30- to 60-minute diagnostic interview conducted in accordance with DSM-5 criteria. The line of schizophrenia refers to schizophrenia spectrum disorders as defined in the DSM-5. Mood disorders corresponds to depressive disorder and bipolar and related disorders; anxiety corresponds to anxiety disorders; and substance refers to substance use disorders, substance intoxication, or substance withdrawal.

We further examined data from emergency department patients who received psychiatric consultations at a tertiary medical centre in Taipei between 2015 and 2023 (*N* = 9548). Similar upward trends were observed: psychiatric emergency visits increased by 530% among individuals aged 0–14 years, 307.4% in those aged 15–24 years, 141.5% in the 25–44 group, 109.4% in the 45–64 group, and 151.4% in the >65 group. Notably, the increase in reported suicide risk (241%) exceeded that of both suicide mortality (108%) and psychiatric emergency visits (131.3%) among males aged 0–24 years (Supplementary Figures 2 & 3). The ratio of growth in psychiatric emergency visits to reported suicide cases was higher in females (1.29) than in males (0.60), suggesting that young males may exhibit lower treatment-seeking behaviours, consistent with previous findings (Rhodes et al., 2013).

To investigate diagnostic contributors to the rising suicide rates among males, we analysed diagnostic trends in psychiatric emergency visits between 2015 and 2023. The increase was primarily driven by substance-related disorders (345% growth) and mood disorders (197% growth) (Figure. 1). While the proportion of mood disorders among all visits remained stable (37.3% in 2015 vs. 38.7% in 2023), the proportion of substance-related disorders rose substantially (from 13.7% in 2015 to 24.9% in 2023).

In comparison to the steady increase in psychiatric emergency visits reported from 1988 to 2014 (Chen et al., 2022), our findings indicate a more rapid escalation from 2015 to 2023, particularly among females and individuals under the age of 25. Diagnostic trends suggest that, despite rising substance- and mood-related disorders, psychiatric service utilisation has increased among female youths but not among male youths, a pattern consistent with other East Asian countries (Hashimoto et al., 2022). As highlighted internationally, youth suicide prevention strategies emphasising timely intervention and assertive follow-up have proven effective in reversing rising suicide trends (Romeu, 2025).

In summary, our findings from Taiwan complement and extend the results of Rice et al., reinforcing the urgent need to address the growing crisis of suicide in male youth. Future prevention strategies should particularly target substance use disorders and mood disorders, with attention to sex-specific differences in treatment-seeking behaviour.

## Supporting information

Chen and Liu supplementary material Chen and Liu supplementary material

## Supplementary material

The supplementary material for this article can be found at https://doi.org/10.1017/neu.2025.10048.
