# Supplementary material for: Response to ‘International trends in male youth suicide and suicidal behaviour’
Source: Acta Neuropsychiatr. 2025 Dec 17;38:e1. doi: 10.1017/neu.2025.10048 (PMC13130244; doi:10.1017/neu.2025.10048)
Supplement: Chen and Liu supplementary material [file S0924270825100483sup001.docx]

**Youth Psychiatric Emergencies in the Context of Rising Suicide Risk: Diagnostic and Help-Seeking Trends in Taiwan, 2015–2023**

Chi Chen, M.D., MSc.^1^, Chun-I Liu, M.D^2^

^1^Department of Addiction Sciences, Taipei City Psychiatric Center, Taipei City Hospital, Taipei City, Taiwan.

^2^Department of Psychiatry, National Taiwan University Hospital, 10002 Taipei City, Taiwan


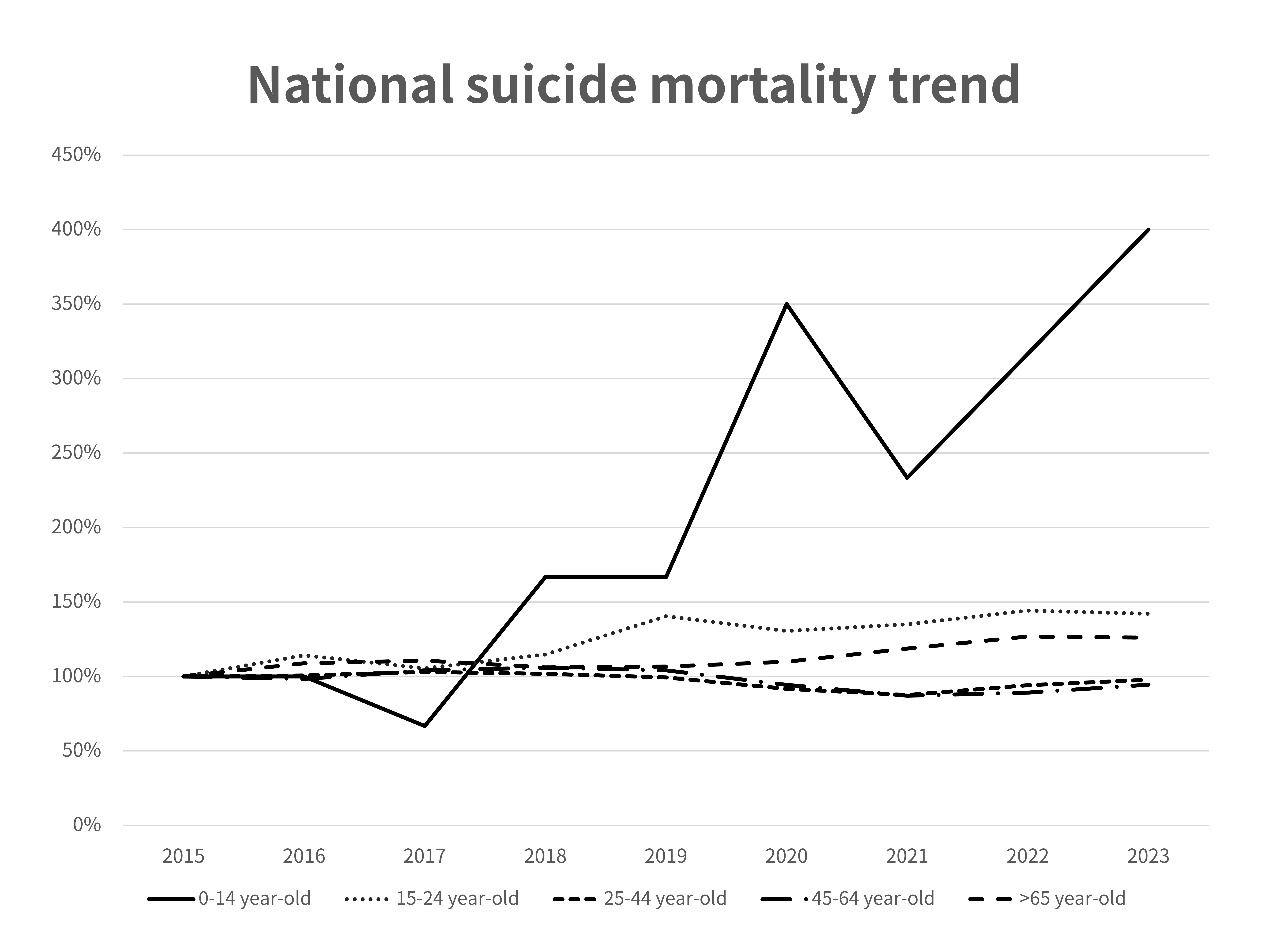


Supplementary Figure 1. National suicide trend from 2015-2023 (the data in 2015 was set as baseline, 100%).

Supplementary Figure 2. National suicide trend and emergent psychiatric visit in a capital tertiary medical center among male aged 0-24 years.

Supplementary Figure 3. National suicide trend and emergent psychiatric visit in a capital tertiary medical center among female aged 0-24 years.
